# Monitoring load, wellness, and psychological variables in female and male youth national team football players during international and domestic playing periods

**DOI:** 10.3389/fspor.2023.1197766

**Published:** 2023-12-14

**Authors:** Thomas Rostgaard Andersen, Bennet Kästner, Mathias Arvig, Carsten Hvid Larsen, Esben Elholm Madsen

**Affiliations:** ^1^ Department of Sports Science and Clinical Biomechanics, University of Southern Denmark, Odense, Denmark; ^2^ Danish Football Association, Brondby, Denmark

**Keywords:** mental health, Hooper index, well-being, stress, training load, monotony, strain, elite

## Abstract

**Aim:**

To study differences in total load exposure, wellness, and psychological variables in youth female (*N *= 19) and male (*N *= 20) national team football players during domestic and international playing periods, respectively.

**Procedures:**

The players filled out questionnaires on well-being, stress, and resilience before and after both playing periods lasting 8 days each. The Hooper index was used to monitor daily wellness levels during both playing periods. The number of training sessions and matches were recorded, and the session rating of perceived exertion was collected. Training load, monotony, and strain were calculated. Daily measurements were used to evaluate in-period changes, and composite scores were used to describe differences between periods.

**Results:**

The international compared to the domestic playing period was for both groups characterized by more matches played, longer field training session durations, and of fewer gym-based sessions (*P *< 0.05). The male players increased total exposure time (25%; *P *< 0.05), monotony (*P *< 0.001), and strain (*P *< 0.001), which was not changed in the female players. Well-being decreased (*P *< 0.05) during the international playing period in male players. Stress levels were higher (*P *< 0.05) for both genders during the international compared to the domestic playing period. During the international playing period, positive correlations were found between the initial levels of stress, and the change in stress (*P *= 0.03; *r*^2 ^= 0.12), and between the changes in total load and changes in well-being (*P *= 0.02; *r*^2 ^= 0.12), whereas a negative correlation was found between the changes in wellness and stress (*P *= 0.03; *r*^2 ^= 0.14).

**Conclusion:**

A playing period characterized by increased match focus, longer field training sessions, and fewer gym-based training activities may lead to changes in the physical and mental profiles of youth national team football players. Alterations to load exposure and wellness may influence mental health. Players with high initial stress levels may be subjected to greater changes compared to other players. Sports scientists and medical staff may benefit from initiating structured monitoring systems to track alterations in physical load and mental health in youth national team players.

## Introduction

The demands of elite-level football have been shown to increase over time (1, 2), and is expected to continuously increase for both female and male players (3, 4). The potential combination of increased physical strain (e.g., more high-intensity training sessions and matches) and psychological stress (e.g., internal/external psychological pressure and intensified travel schedules) may lead to an increased risk of injury (5), as well as a decrease in mental health and well-being (6). In young talented players, new trends indicate that future injury patterns (7), match and training structures (8), as well as elite sports context (9), will be comparable to those of adult elite-level players. Hence, the needs for balancing match and training loads, to ensure optimal recovery, and promote mental health will require an intensified focus also for young players (10).

Physical load may simplistically be divided into external load (e.g., training/match frequency and duration, distance covered in different movement intensity categories etc.) and the associated internal response load (e.g., heart rate, perception of effort). Long- as well as short-term increases in internal load have been shown to be associated with over-training (11), increased risk of injury (12), and increased need for rest and recovery (13). Similarly, sudden changes in normal routines (e.g., extensive travelling, fixture congestion etc.) may negatively influence psychological state variables such as well-being or perceived stress levels (14). Psychological states can be described as characteristic patterns of thinking, feeling, and behaving in a concrete situation at a specific moment in time (15). These can relatively easily be monitored through questionnaires or interviews. The prevalence of mental health problems and mental illness is widespread and increasing among young people (16). In elite sports, athletes are pushing their physical and mental boundaries to enhance performance, and the consequences may be adverse (17). Recently, a study assessed the prevalence of depressive symptoms in Danish elite athletes participating in individual as well as team sports. The study found that female athletes were over-represented in the high-risk depression group (18). Interestingly, a recent study from the Danish premier league also showed, that high resilience (described as the ability to bounce back in times of adversity) may act as a protective factor for mental health. Hence, the level of resilience may potentially impact players' ability to perform and be ready for practice under difficult conditions such as during period with high physical and mental loading (19).

High physical and mental demands are frequently observed in football. Especially during congested match fixtures (20), which is common in professional adult football (21). This may also be the case for international level youth players, who frequently participate in national team training camps or competitions during the playing season (typically on UEFA international dates, 4–7 times/year). Previous research on match fixture congestion in football has mostly focused on adult female and male adult players separately, and with an exclusive focus on changes in either recovery, physical, technical, tactical, cognitive, or mental performance, respectively. Only a few studies [e.g., (20)], have attempted to include measures of changes in physical and mental performance simultaneously.

Therefore, the present study aimed to investigate physical exposure, wellness, and psychological variables in female and male youth national team football players during a domestic playing period with low match frequency in comparison to an international playing period with match fixture congestion.

## Materials and methods

### Participants

Forty-eight female and male international level youth football players selected for the Danish female (FU19) or male (MU19) under-19 national teams were invited to participate in the present study. Inclusion criteria were selected as: female or male youth elite football player selected for the national team roster from August 1st to Nov 30th^,^ 2021. Exclusion criteria were selected as injury or illness resulting in training or match absence during the investigation period, and failure to report data according to the investigation protocol. Of the eligible players, one player declined to participate, and seven players (FU19 = 3; MU19 = 4) accepted to participate but dropped out prior the investigation or during the domestically based monitoring period due to injuries. One player was excluded due to failure to report data. In total, 39 (FU19 = 19; MU19 = 20) players were included in the final analyses. The age of the included players ranged from 16 to 18 years with an average age of 17.9 ± 0.6 years (mean ± SD) and 18.3 ± 0.4 years for the female and male players, respectively. Individual playing positions were distributed as goalkeepers (*N = *4, 10%), defenders (*N = *8, 20%), midfielders (*N = *16, 40%), and strikers (*N = *12, 25%).

### Procedures

The present investigation was conducted as an integrated part of the overall team preparation leading up to the UEFA 2021 European qualifying rounds for FU19 or MU19. The players were separately monitored during a national team training camp on UEFA international playing dates (international playing period; IPP) lasting eight days as well as during a domestic playing period (DPP) of similar duration. Player resilience, perceived stress, and well-being were monitored simultaneously pre- and post-to the IPP and DPP, respectively. During IPP the FU19 played three matches (2 wins—1 draw), and MU19 played two matches (2 wins). On each day during IPP and DPP, the players filled out a five-point Hooper questionnaire in the morning before breakfast to monitor recovery and wellness. The number of training sessions and matches during IPP and DPP, respectively, was recorded separately for all players. In addition, the perceived intensity was individually assessed after each training or match activity. The duration of the activity was registered. The players were familiarized with all data collection procedures prior to the beginning of the investigation. During IPP an DPP, dedicated national team staff members were responsible for all player monitoring procedures, with the data during IPP being collected on-site, and with the data during DPP being transferred to the data collector electronically via applications (22, 23).

### Measurements

#### The Connor-Davidson resilience scale (CD-RISC-10)

The Connor-Davidson Resilience Scale [CD-RISC-10; Connor and Davidson (24)] was used to measure the ability to cope with adversity. On a Likert-scale ranging from 0 (not true at all) to 4 (true nearly all the time), the players were asked to think about the last month and answer 10 questions (e.g., “I am able to adapt when changes occur” and “I am not easily discouraged by failure”). A total sum score was calculated (range: 0–40) with higher scores indicating greater resilience (*α *= 0.73). A composite score of pre- and post-measurements was calculated to represent the average resilience of MU19 and FU19 during IPP and DPP, respectively. The CD-RISC-10 has previously been shown to produce adequate levels of internal consistency within a sport context (25).

#### Perceived stress scale (PSS-10)

The COHEN Perceived Stress Scale [PSS-10; Cohen, Kamarck (26)] was used to measure levels of perceived stress. On a Likert-scale ranging from 0 (never) to 4 (very often), the players were asked to think about the last month and answer 10 questions (e.g., “in the last month, how often have you felt nervous and “stressed”?” and “In the last month, how often have you felt that things were going your way?”). A PSS-10 score was calculated by summation across all 10 items after inverting the scores on the four positive items (items 6, 7, 8, and 9) (*α *= 0.82). A score between 0 and 13 corresponded to a person who “knows how to manage stress”. A score between 14 and 26 corresponded to a person who “generally knows how to cope with stress”. A score between 27 and 40 corresponded to a person where life is considered a “perpetual threat”. A composite score of pre- and post-measurements was calculated to represent the average stress level of MU19 and FU19 during IPP and DPP, respectively. The PSS-10 has previously been shown to produce adequate levels of internal consistency within a sport contexts (*α*'s = 0.82) (27).

#### The world health organization-5 index (WHO-5)

The World Health Organization-5 index [WHO-5; Organization (28)] was used to measure player well-being status. On a Likert-scale ranging from 0 (at no time) to 5 (all the time), the players were asked to answer five questions based on the preceding two weeks (e.g., “I have felt cheerful and in good spirits” and “I have felt active and vigorous”). A WHO-5 score was calculated by the sum of the five answers multiplied by 4 (Range: 0–100) with higher scores indicating good well-being (*α* = 0.76). A composite score of pre- and post-measurements was calculated to represent the average well-being of MU19 and FU19 during IPP and DPP, respectively. The WHO-5 has previously been shown to shown to produce adequate levels of internal consistency within a sport context (*α*'s ≥ 0.70) (29).

#### Recovery and wellness conditions (the Hooper index)

The Hooper index (30) was used to measure player recovery and wellness conditions covering 5 areas (fatigue, sleep quality and time, muscle soreness and psychological wellbeing, respectively). On a Likert-scale ranging from 1 (very, very low or good) to 7 (very, very high or bad) the players rated one question for each subscale: fatigue (e.g., “How fatigued are you?”), sleep quality (e.g., “How was your sleep last night?”), sleep duration (e.g., “How many hours did you sleep last night?”), muscle soreness (e.g., “Please rate your level of muscle soreness”) and psychological [e.g., “How are you feeling psychologically (mentally)?]. The score (min 1–max 7) of each item was evaluated, and a total Hooper score was calculated as the total sum of all item scores (Range: 1–35) (*α *= 0.90). Period mean and a coefficient of variation (C.V. = standard deviation across all individual daily scores divided by the mean multiplied by 100) were calculated for each variable to evaluate variations on total Hooper score and wellness sub-scale scores during IPP and DPP, respectively. The Hooper Index has previously shown adequate levels of internal consistency within a sport context (31).

#### Training sessions and matches

During IPP, the training and match plans for FU19 and MU19, respectively, were organized by the national team staff. During DPP, FU19 and MU19 adhered to the training and match plans organized by their individual clubs. Training sessions were registered as either field or gym sessions, and match participation was reported when a player had been actively involved in international, official league or reserve team matches. Time exposure (training session duration or match playing time) was reported in minutes.

#### Session rating of perceived exertion and training load

Not later than 30 min after cessation of a training session or a match, the players rated their perceived exertion (sRPE) on a 1 (light/easy) to 10 (maximal/hard) point Likert-scale (32). Training load (TL) or match load (ML) was subsequently calculated as TL/ML (A.U.) = sRPE multiplied by activity duration (min). Monotony and strain were calculated as: Monotony = average daily TL divided by the standard deviation of the daily TL monitored during IPP or DPP, respectively, and Strain = Sum of daily TL monitored during IPP or DPP, respectively, multiplied by Monotony. The validity and reliability of the above concepts has previously been reviewed (33).

#### Ethical considerations

Prior to the beginning of the investigation, the players received written and oral information about all study aims as well as associated risks, benefits, and procedures before giving their written and informed consent to participate. If a player at the beginning of the study period was a minor, informed consent was signed by a parent/legal guardian. Participation in the study was voluntary, and a players could withdraw from participation at any time without further notice. The study was reviewed and approved by a local ethics committee (Ethic Committee of Southern Denmark).

#### Calculations and statistical analyses

All variables were tested for normal distribution according to Shapiro-Wilk to justify for the use of parametric statistics. In case of non-normalized distributed data, non-parametric alternatives were applied. Cronbach's alpha was used to test internal consistency reliability of the subscales of CD-RISC-10, PSS-10, WHO-5, and The Hooper Index. A threshold (*α *≥ 0.70) was applied for acceptable reliability. To evaluate within-group changes (FU19 or MU19) between IPP and DPP, respectively, a one-way repeated measures ANOVA was performed separately with stress, resilience, and well-being as the independent variables, and pre- and post-measures for IPP and DPP as the dependent variables. A series of one-way between-subjects ANOVA with repeated measures on wellness (fatigue, sleep quality, sleep duration, muscle soreness, and mental) as the independent variable and with FU19 and MU19 as the grouping variable, was performed for IPP and DPP, respectively. A 2 × 2 contingency table with the application of a Fischer's exact test were applied to evaluate the hypothesis of independence (0-hypothesis) of group (FU19 vs. MU19) and change (increase vs. no change/decrease) during IPP compared to DPP. Correlations were evaluated according to Spearman. For all analyses, *P *< 0.05 was chosen as the level of significance. Data are presented as mean ± SEM unless otherwise stated. All statistical analyses were performed using SPSS Statistics 25 (SPSS, IBM, US).

## Results

In FU19 and MU19, total load exposure was not different (*P* > 0.05) during IPP compared to DPP. FU19 had on average a 10% lower (*P* < 0.05) total number of training sessions and matches during IPP compared to DPP. MU19 on average had a 10% higher (*P* < 0.05) total number of training sessions and matches and a 25% higher (113 ± 26 min; *P* < 0.001) total exposure time during IPP compared to DPP (Table 1). Similar proportions of FU19 and MU19 experienced an increase in total exposure time during IPP compared to DPP (Chi^2^ Fisher Exact; *P* = 0.32).

**Table 1:** Total match and training session exposure in FU19 and MU19 during an IPP and a DPP of equal (8 days) duration.

	Female U19		Male U19	
	DPP	IPP	DPP	IPP
Total exposure				
Total number of training sessions and matches (no.)	8.3 ± 0.4	7.5 ± 0.1*	6.8 ± 0.3	7.5 ± 0.2*
Total exposure time (min)	544 ± 20	556 ± 12	441 ± 25	553 ± 21***
Total load (A.U.)	3,403 ± 158	3,125 ± 98	2,489 ± 171	2,777 ± 120
Monotony (A.U.)	1.52 ± 0.06	1.65 ± 0.06	1.31 ± 0.08	2.07 ± 0.12***
Strain (A.U.)	5,245 ± 406	5,208 ± 290	3,320 ± 361	5,927 ± 540***
Matches				
Average match participation (no.)	1.1 ± 0.1	2.8 ± 0.1***	1.4 ± 0.2	1.9 ± 0.1***
Average match time exposure (min)	51 ± 11	53 ± 7	61 ± 12	52 ± 8
Average match load (A.U.)	515 ± 118	546 ± 80	482 ± 97	385 ± 57
Training sessions				
Average number of training sessions (no.)	7.3 ± 0.4	5.0 ± 0.0***	5.5 ± 0.3	5.6 ± 0.2
Average number of field sessions (no.)	4.9 ± 0.3	4.8 ± 0.1	4.8 ± 0.3	5.6 ± 0.2**
Average number of gym sessions (no.)	2.4 ± 0.4	0.2 ± 0.1***	0.7 ± 0.2	0.0 ± 0.0***
Average session duration (min)	65 ± 9	79 ± 4***	64 ± 9	80 ± 7***
Average training session load (A.U.)	366 ± 67	355 ± 68	312 ± 76	362 ± 57*

* *P* < 0.05;.

** *P* < 0.01;.

*** *P* < 0.01 significantly different DPP vs. IPP within FU19 and MU19, respectively. Numbers are presented as mean ± SEM.

During IPP, the number of match participations increased (*P *< 0.001) compared to DPP in FU19 as well as in MU19. No differences in average total match exposure time and average match load were observed. During IPP, FU19 had a smaller (*P *< 0.001) total number of training sessions and gym-based sessions, respectively, and the average training session duration was longer (Average: + 14 min; *P *< 0.001) compared to DPP. In MU19, the total number of training session was unchanged, whereas the number of field session was greater (*P *< 0.01) and the number of gym-based session was smaller (*P *< 0.001) during IPP compared to DPP. During IPP, the duration of training sessions was longer (Average: + 15 min; *P *< 0.001) in MU19, and the average training session load was higher (*p* < 0.05) compared to DPP (Table 1). Monotony and strain increased (*P *< 0.001) during IPP compared to DPP in MU19 and was unchanged in FU19 (Table 1).

**Figure 1:**
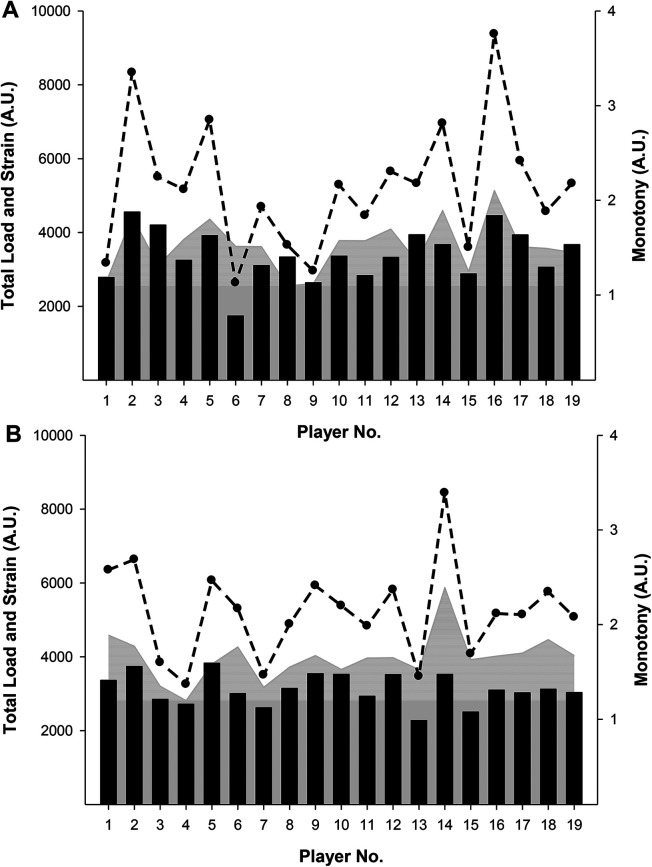
Total load (bars), monotony (grey), and strain (line) in female under-19 national team players during a domestic (**A**) and an international (**B**) playing period. Individual values are presented.

**Figure 2:**
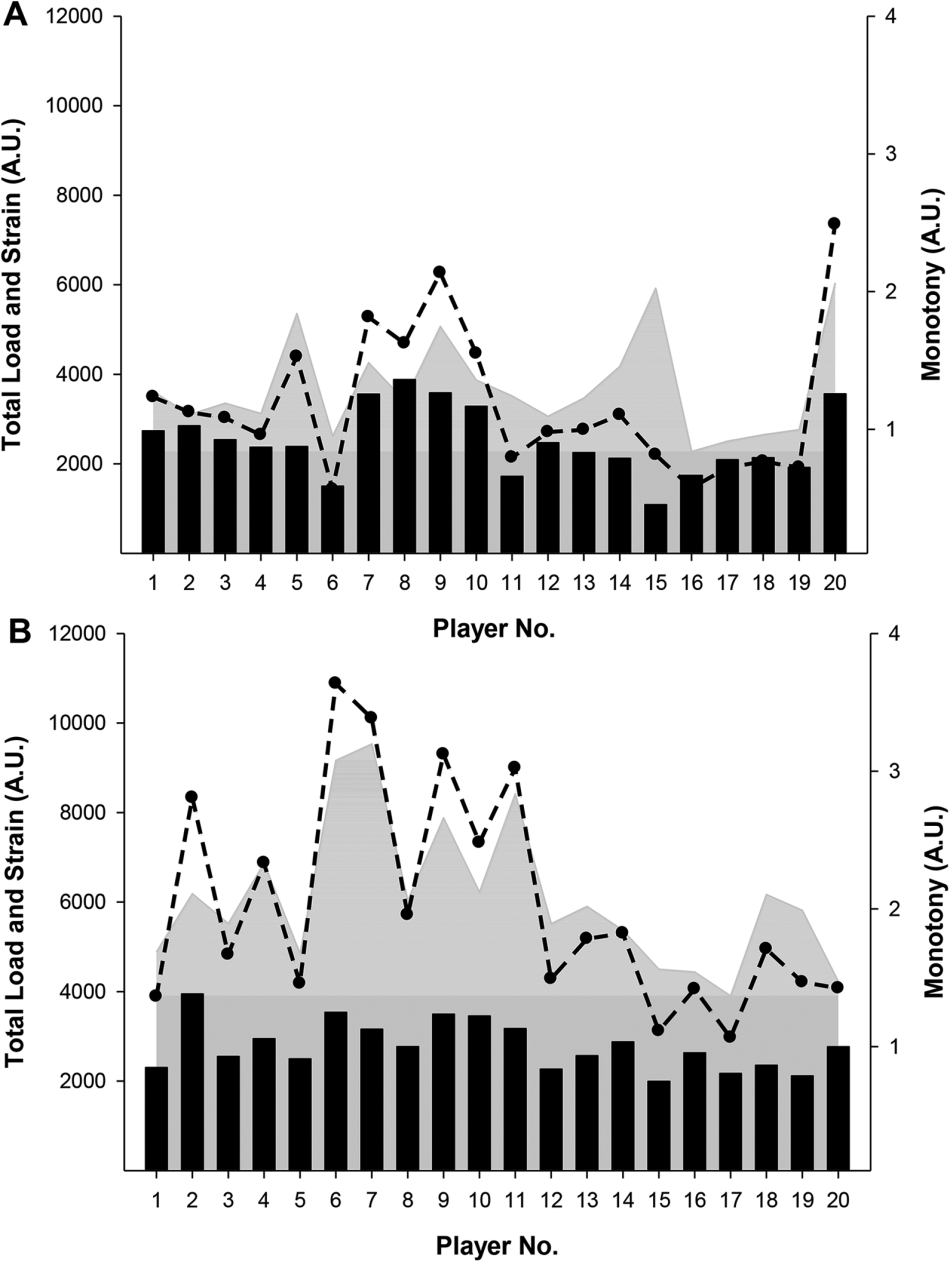
Total load (bars), monotony (grey), and strain (line) in male under-19 national team players during a domestic (**A**) and an international (**B**) playing period. Individual values are presented.

In FU19, marked between-player variations were observed both during IPP and DPP, respectively, for load (range IPP: 2274A.U.-3829A.U.; range DPP: 1740A.U.-4554A.U.), monotony (range IPP: 1.20A.U.-2.39A.U.; range DPP: 1.10A.U-2.10A.U.), and strain (range IPP: 3255U.A-8440A.U; range DPP: 2934A.U.-9375A.U) (Figure 1). In FU19, 37%, 63%, and 53% of the players experienced an increase in load, monotony, and strain, respectively, during IPP compared to DPP. The relative difference between IPP compared to DPP for load, monotony, and strain ranged from −42% to 72%, −25% to 65%, and −44 to 101%, respectively (Figures 1A,B). Similarly in MU19 significant between-player variation was observed during IPP and DPP for load (range IPP: 1995A.U.-3948A.U.; range DPP: 1086A.U.-3880A.U.), monotony (range IPP: 1.37A.U.-3.20A.U.; range DPP: 0.84A.U-2.06A.U.), and strain (range IPP: 2970U.A-10879A.U; range DPP: 1427A.U.-7348A.U) (Figure 2). In MU19, 70%, 85%, and 90% of the players experienced an increase in load, monotony, and strain, respectively, compared to DPP with the relative difference between IPP compared to DPP for load, monotony, and strain ranging from −29% to 136%, −28% to 224%, and −44 to 662%, respectively (Figures 2A,B). More (*p* < 0.05) players in MU19 compared to FU19 experienced an increase in strain during IPP compared to DPP, with no differences observed for load and monotony.

**Figure 3:**
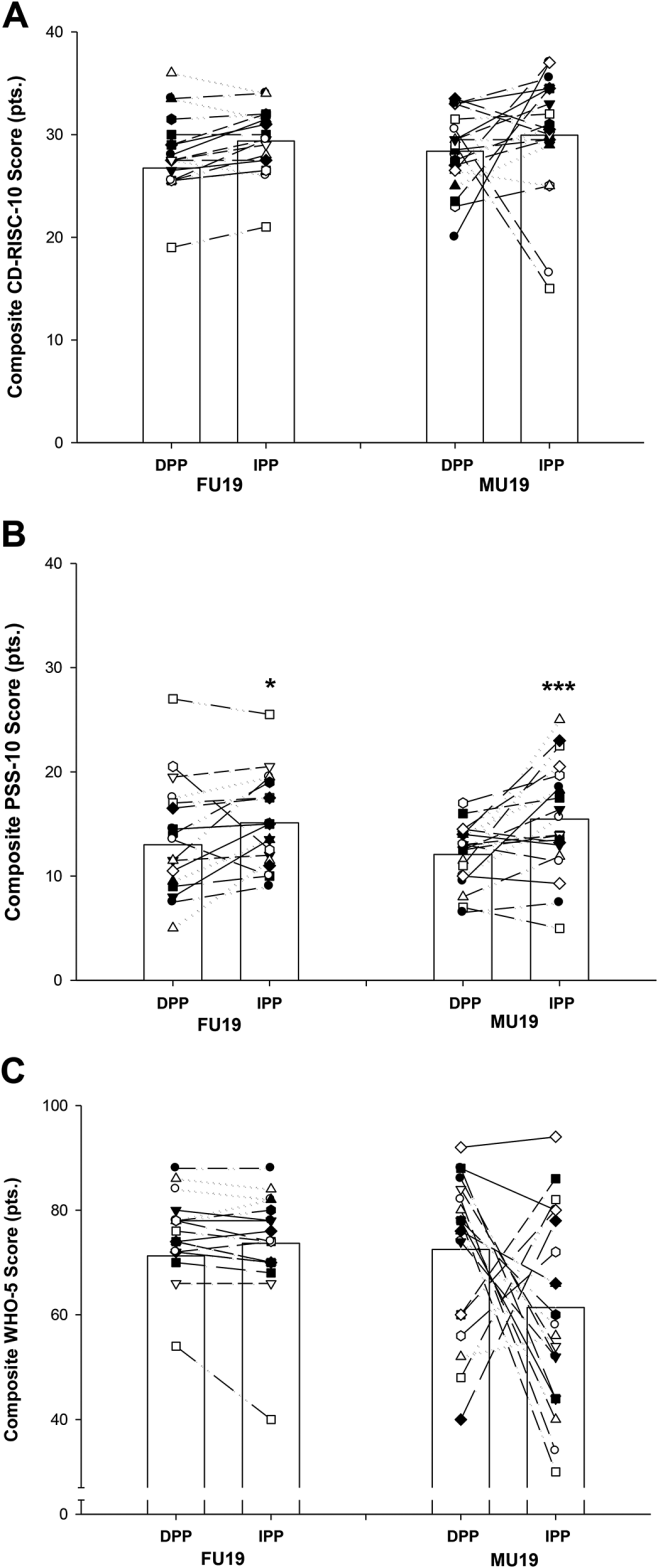
Composite CD-RISC-10 (**A**), PSS-10 (**B**), and WHO-5 (**C**) scores for a domestic (DPP) and an international (IPP) playing period in female (FU19) and male (MU19) national team football players. Bars are mean ± SEM. *P < 0.05. ***P < 0.001. Individual values are presented.

A credible variance indicating heterogeneity was observed between measurements obtained pre- and post- IPP and DPP, respectively, for well-being, stress, and resilience in both FU19 and MU19. Significant group differences were found between FU19 and MU19 in well-being (WHO-5) levels for post-test [F(1.36) = 7.44, *P *< 0.001] during IPP, with no pre- to post-level differences observed for stress and resilience. Composite stress score (PSS-10) analysis revealed a significant difference between IPP and DPP in both FU19 (15.1 ± 1.0 vs. 13.0 ± 1.4; *P *< 0.05) and MU19 (15.5 ± 1.2 vs. 12.1 ± 06; *P *< 0.001), with no differences observed for WHO-5 and CD-RISC-10 (Figure 3). Similar proportions of players in FU19 and MU19, respectively, experienced increases in stress, resilience, and well-being, respectively, during IPP compared to DPP (Figure 3; *P *> 0.05).

Average total Hooper index scores was not different and total Hooper scores C.V. was larger (*P *< 0.05) during IPP compared to DPP for both FU19 and MU19, respectively (Table 2). During IPP, within-subjects differences were found in one of five sub-scale wellness scores [sleep quantity: F(1.1) = 20.615, *P *< 0.001]) indicating significant improvement for the FU19 at the beginning of the IPP. During DPP, no sub-scale wellness score within-subject differences were observed (*P *> 0.05).

**Table 2:** Total Hooper index and sub-scales scores in FU19 and MU19 during an IPP and a DPP of equal (8 days) duration.

	Female U19				Male U19			
	DPP		IPP		DPP		IPP	
	Mean	C.V.	Mean	C.V	Mean	C.V.	Mean	C.V
Total Hooper score	16.4 ± 0.5	16.9 ± 1.5	15.7 ± 0.5	20.9 ± 1.2*	16.2 ± 0.7	16.8 ± 1.4	14.4 ± 0.8	37.2 ± 3.3***
Q1—fatigue	3.4 ± 0.2	19.7 ± 2.0	3.6 ± 0.1	26.3 ± 2.6*	3.4 ± 0.2	28.3 ± 3.9	3.4 ± 0.2	19.8 ± 2.5
Q2—sleep quality	2.9 ± 0.2	27.8 ± 3.3	3.2 ± 0.2	32.5 ± 2.1	3.1 ± 0.2	23.6 ± 2.7	3.4 ± 0.2*	24.9 ± 3.3
Q3—sleep duration	3.6 ± 0.2	29.9 ± 2.8	3.0 ± 0.1**	50.0 ± 1.8***	3.6 ± 0.1	24.2 ± 2.2	3.5 ± 0.2	17.0 ± 2.0*
Q4—muscle soreness	3.5 ± 0.2	21.4 ± 2.8	3.5 ± 0.1	23.1 ± 2.9	3.4 ± 0.2	24.0 ± 2.5	3.2 ± 0.2	20.6 ± 3.6
Q5—mental wellness	3.3 ± 0.2	19.9 ± 2.8	2.9 ± 0.2	22.8 ± 3.7	3.0 ± 0.2	13.7 ± 3.1	2.8 ± 0.2	15.2 ± 3.3

* *P* < 0.05;.

** *P* < 0.01;.

*** *P* < 0.001 significant difference DPP vs. IPP within FU19 and MU19, respectively. Numbers are presented as mean ± SEM and coefficient of variation (C.V.) ±SEM.

In FU19, sub-scale analyses revealed a on average significantly longer (*P *< 0.001) sleep duration during IPP compared to DPP, whereas MU19 reported a decreased (*P* < 0.05) sleep quality during IPP compared to DPP. FU19 and MU19 had a larger (*P* < 0.001) respectively smaller (*P* < 0.05) variation in sleep quality during IPP compared to DPP. In FU19, a larger (*P* < 0.05) variation in fatigue was reported during IPP compared to DPP (Table 2).

**Figure 4:**
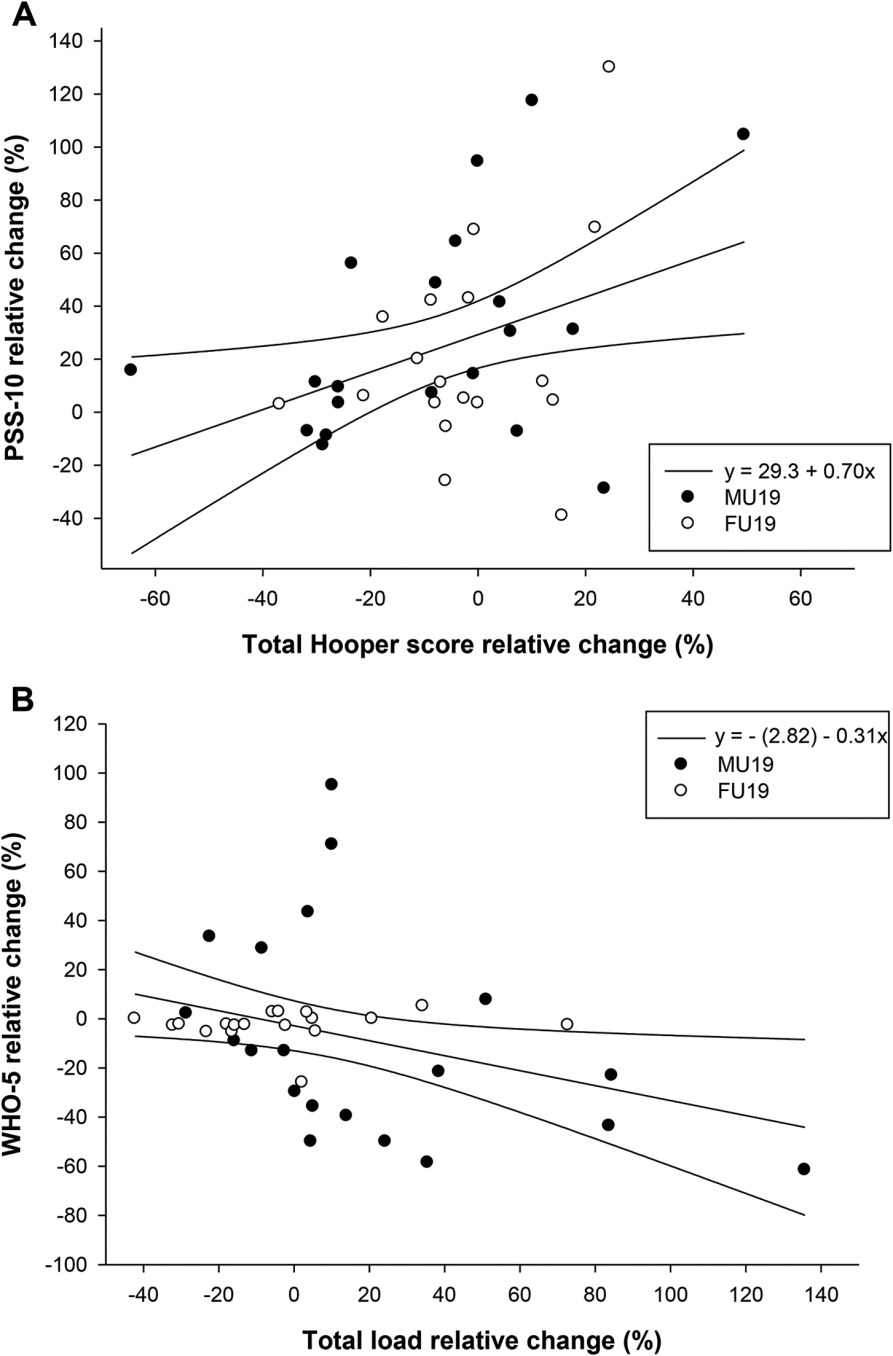
Correlations between relative changes in total Hooper (wellness) score and PSS-10 (stress) (panel **A**; *P* = 0.02; *r* = 0.37), and relative changes in total load (exposure) and wHO-5 (well-being) [panel **B**; *P* = 0.03; *r* = −(0.35)]. Individual values, line of regressions, and 95% confidence intervals are presented.

For all subjects, a significant positive correlation was found between the relative change (IPP vs. DPP) in total Hooper score and the relative change in composite stress (PSS-10; *P *= 0.02; *r*^2 ^= 0.12) as well as between the pre-levels of stress at the beginning of IPP and the change in stress during IPP (*P *= 0.03; *r*^2 ^= 0.12). Furthermore, a negative correlation was found between the relative change in total load and the relative change in composite well-being (WHO-5; *P* = 0.03; *r*^2^ = 0.14), respectively (Figure 4).

## Discussion

The present study investigated changes in physical exposure, wellness, and psychological variables in female and male youth national team football players during international and domestic playing periods. The main findings were, that female and male under-19 national team football players took part in more matches, had longer lasting field training sessions, and had less gym-based training activities during an international period. Also, during an international playing period, the male players had an increase in total exposure time, monotony, and strain. This was not different in the female players. In the male players, well-being was observed to decrease during the international playing period, whereas stress levels were higher for both genders during the international compared to the domestic playing period. Furthermore, moderately strong relationships were observed between the changes in total load and well-being as well as between the changes in wellness and stress, respectively. In addition, a weak-to-moderately strong correlation was found for all players between the initial levels of stress and the change (pre- to post) in stress during an international playing period. Collectively, alterations in load exposure and wellness may influence the mental health of high-level football players. In addition, players with high initial stress levels may be subjected to more unfavourable changes during congested playing periods.

During the international playing period, individual match involvement significantly increased (see Table 1). Congested match-playing periods in professional football, may compromise player recovery profiles compared to non-congested playing periods (defined as 1 or fewer matches within a 7-day period) (34, 35). Specifically for players with longer lasting match involvement (>60 min), substantial fluctuations in load and recovery variables have been observed (36). In our study, the average match playing time and load was moderate for either gender during both observational periods (see Table 1). The most frequent match duration in both genders was full playing time (equivalent to 90–95 min). However, subsequent analysis showed large variations in individual match playing time and load during the two periods (range female: 2–95 min and 10–950 A.U., respectively; range male players: 11–95 min and 100–950 A.U., respectively). Football is an intense intermittent sport, that exerts the entire performance spectrum with high concurrent cardiovascular, metabolic, and musculoskeletal fitness demands (37). Also, football has been shown to lead to physical fatigue (38), and to post-match changes in markers of inflammation, oxidative stress, and performance (39). Also, marked changes in anabolic-to-catabolic ratio (40), increased muscle damage (41), reduced wellness (42), and an increased risk of injury (12) have been reported following match play. Therefore, during both international and domestic playing periods, an augmented focus on specific recovery strategies taking into consideration between players variation (43) may be needed.

National team camps and international playing periods represents a unique context in elite football (44). Typically, limited time exist between the start of a camp and the first match. Similarly, only a few days separate subsequent matches. It has previously been shown that wellness and heart rate variability (31), perceived recovery status (45), and muscle glycogen levels (46) are compromised for several days following a match. Thus, during international playing periods, the ability to carefully accommodate both effective tactical training and sufficient player recovery may influence subsequent game performances. In this study, the average field training session duration was longer during the international compared to the domestic playing period. In addition to a higher game load, this could potentially have resulted in accumulation of fatigue in some players. However, the levels of general fatigue and muscle soreness were unchanged during the international playing period compared to the domestic playing period for both genders (see Table 2). As such, it appears that effective measures were taken by the coaching staff to reduce the risk of inappropriate accumulation of fatigue. This was done by modifying the number of training sessions either on the field or in the gym, reducing general training intensity, and favouring recovery in players with prolonged match involvement (+45 min).

During the international playing period, female players experienced a reduction in the total number of training sessions and matches as well as in total load (−4% ± 26%). In contrast, the male players had an increase in the total number of training sessions and matches. Also, male players experienced average relative increases in total exposure time (33% ± 8%), total load (20% ± 41%), monotony (70% ± 58%), and strain (115% ± 151%), respectively. Studies have shown that the risk of injury may increase between 21% and 49% when training load is increased by ≥15% above the previous week's load (5). Also, monotony, a measure of day-to-day training variability, has been found to be related to the onset of overtraining and poor performance (11, 47). In this study, a considerable proportion of the players on both teams experienced a marked relative increase in load measures in the transition to the international playing period. Specifically, 28% (*N *= 11) of all players experienced a change in total load of 15% or more during the international compared to the domestic playing period. Also, three players had higher strain (Z-score > 2) during the international playing period than the team average. Therefore, several players may have been subjected to an increased risk of injury during the international playing period. However, no time-loss dependent injuries were recorded during the international playing periods or the week immediately following the national team camp in both teams. This indicates that the relative changes and variations in wellness and fatigue, respectively, were within the individual player tolerances. Interestingly, total Hooper index as a marker for wellness and recovery was not affected by match congestion, which is in accordance with previous findings (48).

Both teams had a significant increase in stress levels during the international compared to the domestic period. These findings are consistent with a recent study in elite players investigating mental health and emotional stability throughout a congested season finisher (19). The players in our study also experienced individual fluctuations in well-being levels during the study periods. This indicate that there are inter-individual differences in the way stressful periods are appraised by elite-level football players. The study by Madsen (19) found that resilience was a protective factor for players’ well-being. Thus suggesting, that players with higher resilience levels also experienced higher levels and fluctuations of well-being. This emphasizes the importance of high resilience levels in avoiding negative psychological impacts during stressful training and match periods, as well as during times of adversity (14). In this study, all players were found to have reasonably high levels of resilience throughout the study period, which may have protected some but not all players from unfavourable changes in stress and well-being. Female players' well-being was observed to be stable. In contrast, negative changes in well-being between the international and domestic playing periods were observed in several male players, and with some approaching critical levels (see Figure 3). Resilience was found to be unrelated to changes in stress and well-being, indicating that other factors may have caused the observed changes.

As a novel finding, this study established an associations between the relative change in total wellness and the relative change in composite stress (*r*^2 ^= 0.12). Also, a correlation between the relative change in total load and the relative change in composite well-being was found (*r*^2 ^= 0.14) (see Figure 4). As such, the ability of the coaching staff and medical team to effectively balance individual physical load and effective recovery activities, could potentially positively impact stress levels and well-being (49). Additionally, a significant positive correlation was found between the pre-levels of stress at the beginning of the international playing period and the change in stress during the international playing period (*r*^2 ^= 0.12). This indicate the need for effective monitoring systems to detect players at risk of increased stress. According to the calculated coefficients of determination, changes in total load and wellness accounted for a lesser proportion of the total variance of stress and well-being. However, in high-level football, minor changes in several variables may impact total over-all performance significantly.

During the international playing period, female players experienced an overall increase in sleep duration, while male players experienced a decrease in sleep quality compared to the domestic playing period. Also, at the beginning of the international playing period, female players significantly increased sleep duration (IPP Day-2 vs. IPP Day-1). Several factors may negatively impact sleep. The female players experienced a compressed travelling schedule on IPP Day-1. This may have affected sleep duration in the beginning of the playing period. Also, the female players were not employed on full-time professional contracts. The increase in overall sleep duration for female players may hence be related to increased opportunities to focus on football during the international playing period. The observed decrease in sleep quality in male players and the large variations in sleep quality in both teams during the international playing period may be due to several factors. Specifically, the players were hosted in double rooms, which has been shown to impact sleep in youth elite football players (50). Moreover, match frequency was increased in both teams, potentially resulting in changes in sleep patterns and subsequent in poorer sleep quality during the international playing period (51). Lastly, male players experienced a significant increase in strain during the international playing period. This may have significantly influenced sleep quality (49). Therefore, measures to address the incidence of reduced sleep quality and duration during congested match schedules may advantageously be established. This include optimized travel plans and match schedules, individually tailored training programs, mandatory rest periods, improved sleep hygiene, and an optimized diet (52).

Several limitations apply to the present investigation. Match results in IPP and DPP could have affected resilience, stress, and well-being differently in FU19 and MU19. This is a limitation of this study. Also, during the domestic playing period, training and match schedules were planned by the individual clubs. In contrast, activity schedules during the international playing period were managed by the national team coaching staff. Therefore, training and match exposures were not standardized or controlled making it difficult to address between-team (gender) differences. In addition, the majority of the male players were full-time professionals. Such players have access to multiple club specialist, including sports psychologists and fitness coaches. Generally, the female players had a limited domestic set-up. Female players may consequently have experienced larger unintended variations in study variables during the domestic playing period due to lack of guidance. Furthermore, monitoring data was either collected immediately on-site during the international playing period or electronically reported during the domestic playing period. Subjective measures of exertion may be sensitive to time-delays from the end of physical activity to the time of actual reporting (33). Differences in time-delays between the two observational periods can thus not be excluded, which may have influenced our findings. Also, during the international playing period, all players were equipped with similar GPS-sensors during training sessions and matches. Due to hardware differences between the clubs, this was not possible during the domestic playing period. Large variations between different GPS-systems may exist (53). For this reason, evaluation of changes in external load and its relation to other study variables was not included in the study protocol. This limits the practical application of the findings, and may be a subject to future investigations. Moreover, the data collection in the present study was conducted during two playing periods of shorter (8 days) duration in each team. As such, the data material of the present study was reduced. Inclusion of more observational periods, which has recently been performed by others (48), would increase the robustness of our findings, and reduce the risk of performing type 2 statistical errors. Finally, using self-attributed questionnaires might be subjected to social-desirability bias. As such, players could have answered in a way that portrayed them in a positive light (i.e., answering high on resilience, well-being, and wellness, and low on stress) (54). Despite all staff members being clear upon ethical standards and not sharing results with coaches (potentially providing players with starting positions on the team) this aspect must be considered when interpreting our results.

In brief, this study examined physical exposure, wellness, and psychological variables in youth national team football players. Significant differences between a domestic playing period with low match frequency and an international playing period with match fixture congestion were observed. Specifically, differences in training and match structure between the two periods affected several load, wellness, and psychological variables in a gender-specific manner. Also, the study identified substantial variability in load exposure, recovery, and stress both within and between players during the study period. Finally, the results showed significant associations between changes in measures of recovery and stress, as well as between load and well-being, illustrating the complexity of holistic monitoring in elite team sport athletes.

## Practical implications

Staff members in elite football academies and national teams play vital roles in assisting youth players during challenging times. Therefore, the ability to provide practical recommendations aimed at supporting performance and mental health in youth international level football players may not be underestimated.

Based on our findings, we recommended to incorporate monitoring strategies in clubs and national teams based on quantitative evaluation of mental health-related aspects. Players' psychological states may fluctuate, and therefore, the staff supporting the players could benefit from additional insight into individually assessed mental performance and recovery cycles to initiate customized interventions. This can be accomplished by implementing a stringent procedure for systematically monitoring players' daily wellness using the Hooper Index and/or sleep-tracking devices as recommended by others (55). We suggest using the Hooper Index as part of the daily medical checks. This would allow players a short time to reflect on their wellness levels, providing sports specialists with important information about players' experience of load, recovery, and mental status. A medical check may also serve as an informal platform to individually check-in with the players on several specific personal and performance related domains. Subsequently, the information may be applied to adjust training, individually tailor daily routines, and secure optimal performance. Also, we recommend a continuous tracking of physical strain and exposure. Specifically, football association and clubs may strengthen their shared efforts to exchange individual player information on physical loading across professional barriers prior to and after national team camps and competitive events. This could potentially secure the national team players' continuous ability to perform at their highest possible level and reduce their risk of injury. This might also imply for the mental health condition of the players, as these aspects are important to consider. Based on our results, we also recommend systematic collaborations between sports psychologist working at federation and the club level, respectively, in terms of supporting players in the best possible way in the transition from domestic to international level football.

Based on empirical evidence and our applied experience, gender differences among elite-level team players must be considered. Specifically, female players express greater risk factors of depressive symptoms (18). As such, when players report poor well-being or high stress levels, we recommend addressing these areas via confidential conversations with a team sports psychologist and possibly a team physician. However, recent evidence suggests, that upholding client-psychologist confidentiality may be compromised in elite football (56). This line of research is imperative as limiting help-seeking, stigma toward psychology. Upholding confidentiality between the player, physician, and sports psychologist is pivotal to consider. Especially when setting up effective mental health support systems and assessing if further initiatives or measures are needed. Finally, continuous reviews of individual monitoring protocols are needed to ensure that measures are conducted with a purpose and are not characterized by widespread surveillance of players (57).

## Data Availability

The raw data supporting the conclusions of this article will be made available by the authors, without undue reservation.
